# Open-loop quantum control of small-size networks for high-order cumulants and cross-correlations sensing

**DOI:** 10.1038/s41598-024-67503-x

**Published:** 2024-07-19

**Authors:** Antonio D’Arrigo, Giulia Piccitto, Giuseppe Falci, Elisabetta Paladino

**Affiliations:** 1 Dipartimento di Fisica e Astronomia “Ettore Majorana”, Università di Catania, Via Santa Sofia 64, 95123 Catania, Italy; 2 Dipartimento di Matematica e Informatica, Università di Catania, Viale Andrea Doria, 95125 Catania, Italy; 3 CNR-IMM, Catania (University unit), Consiglio Nazionale delle Ricerche, Via Santa Sofia 64, 95123 Catania, Italy; 4 Istituto Nazionale di Fisica Nucleare, Sezione di Catania, Via Santa Sofia 64, 95123 Catania, Italy

**Keywords:** Nanoscience and technology, Physics

## Abstract

Quantum control techniques are one of the most efficient tools for attaining high-fidelity quantum operations and a convenient approach for quantum sensing and quantum noise spectroscopy. In this work, we investigate dynamical decoupling while processing an entangling two-qubit gate based on an Ising-*xx* interaction, each qubit affected by pure dephasing classical correlated 1/*f*-noises. To evaluate the gate error, we used the Magnus expansion introducing generalized filter functions that describe decoupling while processing and allow us to derive an approximate analytic expression as a hierarchy of nested integrals of noise cumulants. The error is separated in contributions of Gaussian and non-Gaussian noise, with the corresponding generalized filter functions calculated up to the fourth order. By exploiting the properties of selected pulse sequences, we show that it is possible to extract the second-order statistics (spectrum and cross-spectrum) and to highlight non-Gaussian features contained in the fourth-order cumulant. We discuss the applicability of these results to state-of-the-art small networks based on solid-state platforms.

## Introduction

In the current generation of solid-state devices for quantum technologies^1^, environmental noise sets the accuracy limits of quantum gates^2^. Despite the tremendous progress in the last two decades^3–5^, material-inherent noise sources still represent a problem making unreliable even moderate-size quantum circuits. Quantum control techniques^6^ represent one of the most efficient tools to attain high-fidelity quantum operations fulfilling given time and power constraints. Their primary goal is to maintain noise-induced errors below a fault-tolerance threshold required for the efficient implementation of quantum error correction. Dynamical decoupling (DD)^7–9^ is a form of open-loop quantum control whose efficiency has been repeatedly validated in experiments using a variety of platforms^10–14^. The effect of DD can be seen as a noise filtering process^15^ mathematically expressed in terms of (generalized) filter functions (FFs)^16–19^.

From a different perspective, dynamical control can be turned into a tool for quantum sensing (QS) and quantum noise spectroscopy^20^ whereby properly designed pulsed^21–29^ or continuous-control protocols^19,30–35^ allow inferring microscopic information, as noise power spectra. This provides complete statistical information on Gaussian processes whereas the characterization of non-Gaussian fluctuations requires estimating higher-order correlation functions, or polyspectra in Fourier space^36,37^. Discriminating this type of information is of paramount relevance in state-of-the-art devices^33,38,39^ where evidence of microscopic two-level systems either coherently coupled to the quantum circuit or incoherently evolving like random telegraph noise (RTN) processes, has been demonstrated both in spectroscopy and in time-domain measurements^40^. RTN is the archetypical non-Gaussian process and higher-order spectral estimation using a qubit probe under pulsed control^23,37,41^ or via a frame-based control-adapted FF formalism^42^ have been recently demonstrated. Correlated Gaussian processes^43,44^ and RTN^36^ inducing pure dephasing have been investigated via multipulse quantum noise spectroscopy protocols. Collections of RTNs with proper distribution of switching rates are a common model of noise with power spectrum behaving as $1/f^\alpha$ where *f* is the frequency and $\alpha \sim 1$^45^. Inherently microscopic noise sources with 1/*f*-like spectral density and/or non-Gaussian characteristics^40^ represent one of the major problems for quantum state processing of state-of-the-art scalable solid-state qubits^5,40,46–54^. Preservation of entanglement in the presence of RTN or 1/*f* noise via DD protocols has been also proposed^55–60^.

DD of local and spatiotemporal correlated noise sources with 1/*f* or RTN spectrum in an entangling gate is a critical step to achieving high-fidelity two-qubit gates. This issue received so far less attention, despite recent experiments revealing spatial noise correlations^33,61^. DD of a two-transmon gate with a noisy tunable coupler has been recently investigated^52^, pointing out the role of 1/*f* flux noise in the coupler and observing non-Gaussian signatures analogous to those investigated in single qubit gates^62,63^.

In this work, we consider an entangling two-qubit gate based on an Ising-*xx* interaction with strength $\omega _c$. Each qubit is affected by local pure dephasing classical noises with power spectrum $S(f) = A/f$ in the range of frequencies $f \in [f_m, f_M]$ with some degree of correlation^40^ quantified by a non-vanishing cross-spectrum $S_c(f)$ between the random forces. We consider processes characterized by $S_c(f)= \mu \, A/f$, where the parameter $\mu \in [0,1]$ quantifies the strength of the correlations^33,52,64^.

We study DD protocols implemented by sequences of instantaneous pulses acting on each qubit locally and simultaneously designed in a way not to alter the capability of the gate to generate entanglement at a time $t_e= \pi /(2 \omega _c)$. We evaluate the gate error, both in the time and in the frequency domain, using a Magnus expansion technique. For local longitudinal noise, the evolution is exactly mapped to two-level problems with *transverse* coupling to classical noise. By following an approach inspired by^16,17^, we derive an approximate analytic expression for the error as a hierarchy of nested integrals of noise cumulants and FFs, that is the main result of the paper. Depending on the DD sequence and the statistical properties of the noise, the gate error is dominated by contributions of cumulants of different order. Up to the fourth order, we can separate the error in contributions due to Gaussian and non-Gaussian components identifying the corresponding FFs. The different scaling of these terms with the correlation parameter $\mu$ allows the characterization of the noise statistics and cross-correlations. By exploiting the filtering properties of the DD sequences considered, we show that it is possible to extract the second-order statistical properties (spectrum and cross-spectrum) and to highlight non-Gaussian features by the fourth-order cumulant.

The paper is structured as follows. In Section “Results” we briefly introduce the model and the DD protocol. Then we present the main results of the paper: we report the analytical expression for the gate error and we show some numerics on some possible application of this technique for quantum sensing. We discuss these results in Section “Discussion”. We leave technical details to Section “Methods”.

## Results

In this section we present the main results of this work. We start from the analytical expression for the gate error and some related numerical results. Then we present some application of the DD protocol for QS, in particular for detecting non-gaussianity or spatial correlation of the noisy environment.

### The protocol

We start considering a system of two coupled identical qubits in the presence of classical noise described by the Hamiltonian ${{{\mathscr {H}}}}(t) = {\mathscr {H}}_0 + \delta {\mathscr {H}}(t)$ where (units of $\hbar =1$ are chosen)

1 $$\begin{aligned} {\mathscr {H}}_0 = -\frac{\Omega }{2} \, \sigma _{1z} \otimes \mathbbm {1}_{2} -\frac{\Omega }{2} \, \mathbbm {1}_{1} \otimes \sigma _{2z} + \frac{\omega _c}{2} \, \sigma _{1x} \otimes \sigma _{2x}, \quad \delta {\mathscr {H}}(t) = -\frac{z_{1}(t)}{2} \, \sigma _{1z} \otimes \mathbbm {1}_{2} - \frac{z_{2}(t)}{2} \mathbbm {1}_{1} \otimes \sigma _{2z}, \end{aligned}$$

where $\sigma _{\alpha x}$ and $\sigma _{\alpha z}$ are the Pauli operators acting on the qubit $\alpha$, being the logic basis such that $\sigma _{\alpha z} |\pm \rangle _{\alpha } = \mp |\pm \rangle _\alpha$. When the qubits natural frequencies $\Omega$ are much larger than the coupling strength $\omega _c$, the evolution for a time $t_e = \pi /2\omega _c$ under ${\mathscr {H}}_0$ implements a $\sqrt{i-\text {SWAP}}$ two-qubit gate which has been demonstrated on different hardware platforms^2,65–67^. For the sake of presentation, we focus on the effects of local classical noise longitudinally coupled to each qubit i.e. noise enters $\delta {{{\mathscr {H}}}}(t)$ with terms commuting with the individual qubit Hamiltonian and discuss this choice later (see Section “Discussion”). Noise is modeled by two stochastic processes $z_\alpha (t)$ assumed to be of 4-th order stationary and characterized by their power spectra $S_{z_\alpha }(\omega )$ and fourth-order cumulants. Correlations of noises on different qubits are quantified by the cross-spectrum $S_{z_1 z_2}(\omega )$ (see Supplemental Section B). Hereafter we set $\omega _c = 5 \times 10^9 rad/s$ as the energy scale of the system.

Control is operated by applying simultaneously to both qubits a sequence made of an even number of $\pi -$pulses around the $y-$axis of the Bloch sphere as described by the Hamiltonian ${{{\mathscr {H}}}}_C(t)$ in Eq. (11). This protocol is designed to dynamically decouple the system from the noisy environment while executing a two-qubit gate. Indeed, in the asymptotic limit of a large number of pulses, the sequence averages out the diagonal terms of the Hamiltonian while keeping the coupling term $\sigma _{1x} \otimes \sigma _{2x}$. The error in the gate operation is quantified by

2 $$\begin{aligned} \varepsilon \,=\,1-\langle \psi _e|\rho (t_e)|\psi _e\rangle , \end{aligned}$$

where $|\psi _e\rangle$ is the target state of the ideal operation and $\rho (t_e)$ is the actual state at $t=t_e$ obtained from the evolution under the action of the controlled noisy Hamiltonian ${{{\mathscr {H}}}}(t)+{{{\mathscr {H}}}}_C(t)$. The gate infidelity is the maximal error with respect to the initial state $|\psi _0\rangle$.

Under the action of ${{\mathscr {H}}}$, the system evolves in two invariant subspaces (see Supplemental Section A). We focus on the dynamics in the single-excitation subspace $W= \textrm{span}\{|+-\rangle ,|-+\rangle \}$. In the basis of the Bell states $|\beta \rangle = \big [|+-\rangle + (-1)^\beta |-+\rangle \big ]/\sqrt{2}$ for $\beta =1,2$ (see Supplementary Table S1), the projected Hamiltonian reads

3 $$\begin{aligned} {{{\mathscr {H}}}}_{W}(t):= P_W \, {{{\mathscr {H}}}} P_W = -\, {\frac{\omega _c}{2}}\, \tau _z - \frac{z_1(t) - z_2(t)}{2} \,\tau _x , \end{aligned}$$

where $P_W$ are projection operators and $\tau$’s are Pauli matrices, $\tau _z = |1\rangle \langle 1|- |2\rangle \langle 2|$ and $\tau _x = |1\rangle \langle 2| + |2\rangle \langle 1|$. Therefore the effective dynamics in the *W* subspace is governed by a two-state Hamiltonian. The ideal gate generated by $\sigma _{1x} \otimes \sigma _{1x}$ is projected in a two-level unitary of the *W*-subspace which operates as a non-trivial quantum gate. The effective noise enters via the stochastic process $\zeta (t)=z_1(t)-z_2(t)$ which couples by an operator *transverse* to the projected ideal Hamiltonian $P_W \, {{{\mathscr {H}}}}_0 P_W$.

In particular, we study the generation of a maximally entangled state obtained from the initial factorized state $|\psi _0\rangle = |+-\rangle$ by evolving the system in the absence of noise for a time $t_e$

4 $$\begin{aligned} |\psi \rangle _e = e^{-i {\mathscr {H}}_0 t_e} |+-\rangle = \frac{|+-\rangle - i |-+\rangle }{\sqrt{2}}. \end{aligned}$$

In the following, we focus on the error $\varepsilon$ for this operation. This quantity will be used as the output of a QS protocol and it also provides an indicator of the gate fidelity in the *W*-subspace since the chosen $|\psi _0\rangle$ approximately maximizes the gate error for $\zeta \ll \omega _c$.

### Gate error under dynamical control

We consider a control Hamiltonian ${\mathscr {H}}_c$ as in Eq. (11) (Section “Methods”). We notice that the dynamics under this $y-y$ pulse sequence, described by Eq. (14), preserve the invariant subspaces of ${{\mathscr {H}}}$. Therefore, under DD control, the gate error for the operation Eq. (4) does not contain contributions due to leakage from the *W* subspace. We consider three decoupling sequences of 2*n* pulses, namely the periodic (P)^7,8^ the Carr-Purcell (CP)^68^ and the Uhrig (U)^69^ sequences (see Section “Methods” for details). They differ for the times $t_i$s when the pulses are applied, resulting in different noise filters. One of the key results of this work is the following formula expressing the gate error $\varepsilon$ as an expansion in the time-correlations of the noise truncated at the fourth-order, as a function of the system parameters and the number of pulses *n*

5 $$\begin{aligned} \begin{aligned} \varepsilon \,= \varepsilon ^{[2]} + \varepsilon ^{[4]}_g + \varepsilon _{ng}^{[4]} = \,&\int _{-\infty }^{+\infty }\frac{d\omega }{2\pi }\,S_\zeta (\omega ) F_{1}(\omega ,\omega _c,t_e,2n)\, \\ + \,&\int _{-\infty }^{+\infty }\frac{d\omega _1}{2\pi }\,S_\zeta (\omega _1) \int _{-\infty }^{+\infty }\frac{d\omega _2}{2\pi }\,S_\zeta (\omega _2)\, F_{2,g}(\vec \omega _2,\omega _c,t_e,2n)\,\\ +\,&\int _{-\infty }^{+\infty }\frac{d\omega _1}{2\pi }\, \int _{-\infty }^{+\infty }\frac{d\omega _2}{2\pi }\,\int _{-\infty }^{+\infty }\frac{d\omega _3}{2\pi } S_{\zeta 3}(\omega _1,\omega _2,\omega _3)\, F_{2,ng}(\vec \omega _3,\omega _c,t_e,2n) \,, \end{aligned} \end{aligned}$$

with the analytic form for the FFs $F_i(\omega ,\omega _c,t_e,2n)$ in Eq. (26). The second-order $\varepsilon ^{[2]}$ depends on the power spectrum $S_\zeta (\omega )$ of the noise $\zeta (t)$. The latter is the sum of the power spectra of each physical process $z_\alpha (t)$ and of their cross-correlation (see Supplemental Section B), $S_\zeta (\omega )= S_{z_1}(\omega ) +S_{z_2}(\omega ) - 2 S_{z_1 z_2} (\omega )$. The fourth-order $\varepsilon ^{[4]}$ can be written in general as the sum of a Gaussian ($\varepsilon ^{[4]}_g$, second line) and a non-Gaussian ($\epsilon _{ng}^{[4]}$, third line) contribution. This latter depends on the trispectrum $S_{\zeta 3}(\omega _1,\omega _2,\omega _3)\,$ which is the Fourier transform of the (stationary) fourth-order cumulant of $\zeta (t)$.

For a fixed duration $t_e$ of the gate operation, we analyze the dynamics under ${{{\mathscr {H}}}}_C(t)$ for the three different sequences (P), (CP) and (U). Information on the pulse-sequence enters Eq. (5) via the FFs $F_i(\omega ,\omega _c,t_e,2n)$. Notice that our $F_i$s generalize the FFs used for standard DD and QS of longitudinal noise^10^. In our case a non-trivial gate operation is performed during the DD sequences, thus noise operators do not commute anymore with the Hamiltonian $P_W \, {{{\mathscr {H}}}}_0 P_W$. The expression of the generalized FFs, which in our case explicitly depend on the coupling $\omega _c$, has been evaluated by exploiting the Magnus expansion of the evolution operator (see Supplemental Sections D and E).

**Figure 1:**
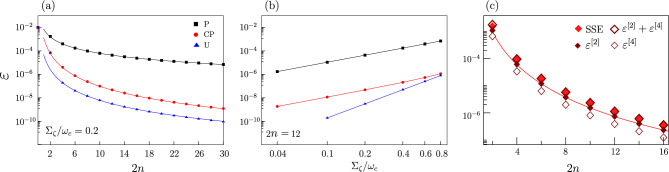
(**a**) Gate error $\varepsilon$ for P (black), CP (red), and U (blue) sequences as a function of the number of pulses 2*n* for fixed gate time $t_e = \pi /2\omega _c$ , with $\omega _c = 5 \times 10^9 rad/sec$ and noise amplitude $\Sigma _\zeta =0.2\omega _c$. Symbols are data from the numerical solution of the SSE, the filled lines are the analytical expressions Eq. (6) for P and CP sequences, the dashed line is the fit in Eq. (7) for U sequence. (**b**) Gate error $\varepsilon$ for the three sequences as a function of $\Sigma _\zeta /\omega _c$ for $2n = 12$. The filled lines are guides to the eyes. Notice the logarithmic scale on both axes. (**c**) different contributions to $\varepsilon$ for the CP sequence for $\Sigma _\zeta = 0.8 \omega _c$: light red filled diamonds are the numerical solution of the SSE, large dark red diamonds are the error $\varepsilon$ Eq. (5), the continuous red line is Eq. (6). The second and fourth-order contributions to the error correspond to the dark red-filled diamonds and small dark red diamonds respectively. Similar behavior for the Uhrig sequences is shown in Fig. 2.

A first insight into the problem is gained by considering Gaussian quasi-static (qs) noise with variance $\Sigma _\zeta ^2$. In this limit, only frequencies much lower than $\omega _c$ enter Eq. (5), thus the power spectrum can be approximated by $S_\zeta (\omega ) = 2 \pi \Sigma ^2_\zeta \delta (\omega )$. In Fig. 1 we show $\varepsilon$ for the pulse sequences under study and various $\Sigma _\zeta$. The symbols are the numerical solution of the stochastic Schrödinger equation (SSE). The filled lines in panel (a) and panel (b) are the following analytical expressions

6 $$\begin{aligned} \varepsilon _{qs}^{(P)} \simeq \frac{\pi ^2}{2^6}\left( \frac{\Sigma _\zeta }{\omega _c}\right) ^2\frac{1}{n^2} \quad , \quad \varepsilon _{qs}^{(CP)} \simeq \frac{\pi ^4}{2^{12}}\left( \frac{\Sigma _\zeta }{\omega _c}\right) ^2\frac{1}{n^4} , \end{aligned}$$

derived from Eq. (5) by substituting the power spectrum. We notice that there is an excellent agreement between numerics and analytical approximations. Both sequences suppress noise for increasing pulse rate and decreasing ratio ${\Sigma _\zeta }/{\omega _c}$, in agreement with the analogy between DD and the Zeno-effect^70^. Moreover, even though both errors scale quadratically with $\Sigma _\zeta$, the CP ($\propto 1/n^4$) produces, for increasing pulse rate, stronger error suppression than the P sequence ($\propto 1/n^2$). The numerical analysis for the CP sequence suggests that, for $\Sigma _\zeta > \omega _c$, the second and the fourth-order terms contribute $\varepsilon$ with comparable magnitude. This is shown in panel (c) where we plot the second-order and the fourth-order contribution, $\varepsilon _g$ and the solution of the SSE, for $\Sigma _\zeta = 0.8 \omega _c$.

The approximate analytic behaviour for the U sequences (filled lines in Fig. 1c) is derived by fitting, for $n > 4$, the SSE numerical result as $\varepsilon (n) \sim an^b$, finding

7 $$\begin{aligned} \varepsilon _{qs}^{(U)} {\sim \frac{\pi ^4}{2^{12}}} \left( \frac{\Sigma _\zeta }{\omega _c}\right) ^4\frac{1}{n^{{3.75}}}. \end{aligned}$$

The dependence on $\Sigma _\zeta ^4$ suggests that the Uhrig filter fully cancels the contribution of the second-order time-correlation function during processing in the presence of transversal effective noise.

**Figure 2:**
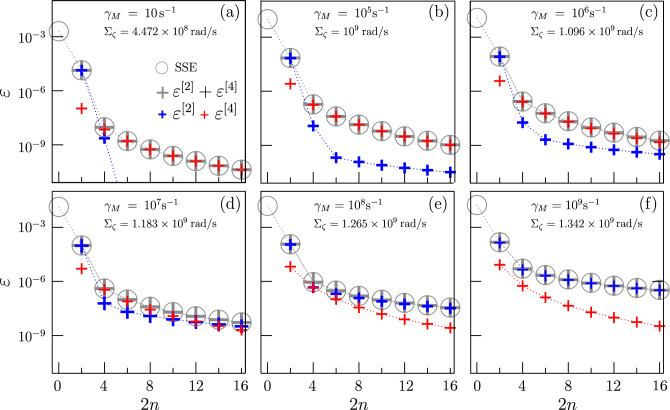
Gate error for the U sequence as a function of 2*n* for a Gaussian 1/*f* noise with fixed low-frequency cutoff $\gamma _m=1s^{-1}$. Different panels refer to different upper cut-off $\gamma _M$ and $\Sigma _\zeta$ is chosen such as to have the same integrated power spectrum for any $\gamma _M$ (parameters in panel (**b**) correspond to typical values of charge noise in superconducting qubits). In each panel, open circles are the solutions of the SSE equation, grey crosses give the error of Eq. (5), while blue and red crosses represent the 2nd and the 4th order Gaussian contributions, respectively. The Uhrig sequence practically cancels the 2nd order contribution for $\gamma \le 10^{6} s^{-1}$.

Remarkably, the same results can be derived for the case of long-time correlated noise. In Fig. 2 we report the gate error $$\varepsilon$$ in the presence of noise with spectrum $$S_\zeta (\omega )=A/\omega$$ having fixed low-frequency cut-off $$\gamma _m=1s^{-1}$$ and varying the upper cut-off $$\gamma _M$$ = $10, 10^5, 10^6, 10^7, 10^8, 10^9 text{s}^{-1}$ for panels (**a**), (**b**), (**c**), (**d**), (**e**), (**f**), respectively . For any $$\gamma _M$$ we fix $$\Sigma _\zeta$$ in order to keep fixed the integrated power spectrum. Open circles are the solution of the SSE equation, grey crosses are obtained by Eq. (5), and blue and red crosses represent respectively the second and the fourth-order contribution in Eq. (5). The analytical approximation Eq. (5) and the numerical SSE are in agreement also in this case. This result assesses the ability of the DD procedure to suppress noise at $$\omega _c \ne 0$$ and yields also in this case the Zeno effect scenario. For low-frequencies noise ($$\gamma _M \le 10^6 \ \text {s}^{-1}$$) the error is dominated by the fourth-order term, while the situation is reversed and the second-order contribution becomes dominant, as soon as high-frequency noise enters the game, i.e. $$\gamma \ge 10^{-8} s^{-1}$$. Sensitivity to the control of decoherence induced by different noise decades can be used as a QS tool providing spectral information on the noise. In the particular example of Fig. 2 the comparison between experimentally measured contribution to the gate error can provide information on the dominant frequency components of the noisy environment.

### Filter functions and quantum sensing

**Figure 3:**
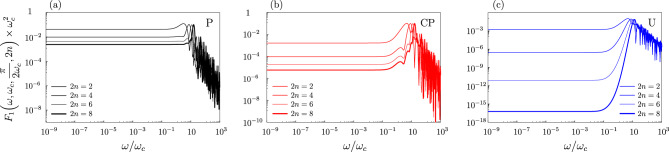
Plots of the second order FF $F_{1}^{}(\omega ,\omega _c,t_e,2n)\times \omega _c^2$, Eq. (26), with $t_e=\pi /(2\omega _c)$, as a function of $\omega /\omega _c$. Different colors refer to different sequences: P (black, panel (**a**), CP (red, panel (**b**)) and U (blue, panel (**c**)). In each figure, the curves refer to (from top to bottom) $2n=2,\, 4,\,6,\, 8$. The U-filter suppresses low-frequency noise by several orders of magnitude already with a small number of pulses.

Standard FFs^71^ can be designed to increase the protection of coherence from longitudinal noise using DD techniques^10^. Moreover, modulation of properly designed filters can be used to detect specific characteristics of noisy environments (e.g. the non-Gaussianity), making it a powerful tool for QS of noise^20^. For example, a possible application is that of using a single-quibit to experimentally characterize the longitudinal noise^22–26,72^. In this section, we investigate the behaviour of our generalized FFs. The main result suggests that the pulse sequences can provide distinct signatures of the local dephasing bath such as non-Gaussianity and spatial correlations for 1/*f*-like noise. In Fig. 3 we show $F_{1}(\omega ,\omega _c,t_e,2n)$ for $t_e=\pi /(2\omega _c)$ appearing in Eq. (25), for the three pulse sequences introduced previously. Regardless of the sequence considered, the filter has a maximum at $\omega \sim \omega _c$, which becomes sharper for increasing the number of pulses *n*.

For frequencies $\omega \ll \omega _c$, i.e. those of interest for the perspective of canceling low-frequency noise, the filter has a plateau whose magnitude decreases with increasing the number of applied pulses. This result suggests that our generalized FFs are suitable for this purpose. The ratio between the value at the peak frequency and the magnitude of the low plateau for $n \ge 3$ is moderate for the P generalized filter, but it may be very large for the U one.

**Figure 4:**
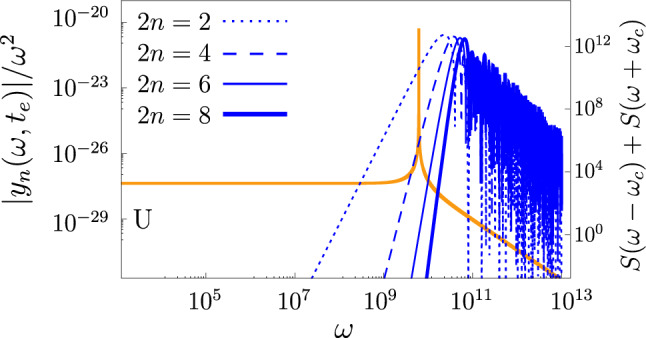
The modified 1/*f* power spectrum $S(\omega -\omega _c)+S(\omega +\omega _c)$ (orange curve) in Eq. (8), $\gamma _{m}= 10^0$ s$^{-1}$, $\gamma _{M}= 10^6$ s$^{-1}$ and $\Sigma _\zeta = 4\cdot 10^8$ s$^{-1}$. It behaves as a narrow filter for the second-order $F_1(\omega ,t_e,2n)$ weakening the impact of low-frequencies. Indeed, due to the small overlap of the blue and orange curves, the error $\varepsilon ^{[2]}$ as given by the integral Eq. (8), is suppressed. For the Uhrig filter $F_1^U(\omega , t_e,2n)$ (blue curves) and $t_e = \pi /(2 \omega _c)$, frequencies up to $\sim ~ 2 \pi /t_e$ are suppressed already for $2n=6$.

In particular, the U protocol efficiently filters out low-frequency noise in second order. As a consequence, a relatively small number of pulses is enough to suppress the error $\varepsilon$ by several orders of magnitude. To clarify this property we first observe that the behavior of the generalized second-order FFs $F_1(\omega ,\omega _c,t_e, 2n)$ shown in Fig. 3 is significantly different than that of the standard ones $F_1(\omega ,t_e, 2n)$ for pure-dephasing noise, the blue line in Fig. 4. We can make a connection by rewriting the error $\varepsilon ^{[2]}$ in Eq. (5) in terms of the standard filters

8 $$\begin{aligned} \varepsilon ^{[2]} \,=\,\frac{1}{16}\int _{-\infty }^{+\infty }\frac{d\omega }{2\pi } \Big [ S_{}(\omega -\omega _c) + S_{}(\omega +\omega _c)\Big ] \, F_1(\omega ,t_e,2n) . \end{aligned}$$

The sum of the shifted spectra $S_{}(\omega -\omega _c) + S_{}(\omega +\omega _c)$ shown in orange in Fig. 4, has a sharp peak at $\omega _c$ while at smaller frequencies it exhibits a plateau whose value is orders of magnitude smaller than the original 1/*f* spectrum $S(\omega )$. Thus Eq. 8 and Fig. 4 suggest that the system dynamics during the gate operation acts as a narrow filter at frequency $\omega _c$ for the standard $F_1(\omega ,t_e,2n).$In particular, by using the Uhrig sequence we can leverage the properties of the corresponding standard $F_1^U(\omega ,t_e,2n) ={\big |y_n(\omega ,t_e)\big |^2}/{\omega ^2}$ (see Section “Filter function formalism”), which describes decoupling in idle time intervals, to achieve during processing a suppression of environmental noise up to frequencies $\sim 2 \pi /t_e = 4 \omega _c$ already with a small number of pulses. Notice that larger *n*’s are required to achieve the same suppression by the P and the CP sequences (see Fig. S1). For an increasing number of pulses, the rate of change of $F_1^U(\omega ,t_e,2n)$ for $\omega \lesssim 2 \pi /t_e$ becomes very small and the gate error is due to the fourth-order statistical properties of the noise in agreement with the results in Fig. 2.

**Figure 5:**
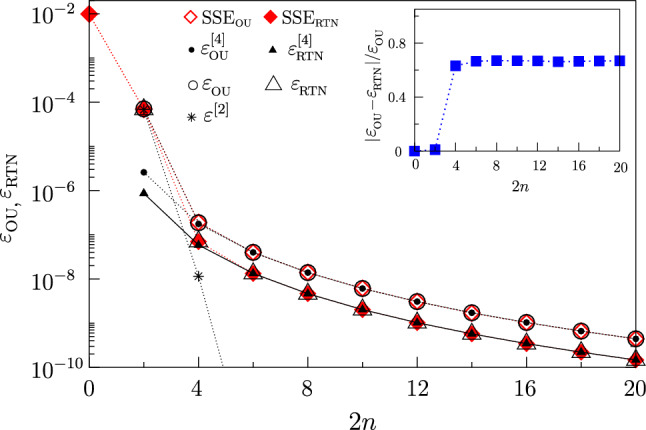
Gate error under Uhrig DD at $t_e$ as a function *n*. Symbols are the solution of the SSE: for Gaussian noise (OU, open diamonds) and non-Gaussian noise (RTN, filled diamonds). Both processes have zero average and the same variance, $\Sigma _\zeta /\omega _c=0.2$. The Gaussian noise is obtained by an ensemble of 256 RTNs with the same $\gamma = 1$ s$^{-1}$, whereas non-Gaussian noise is produced by a single RTN with $\gamma = 1$ s$^{-1}$. The error given by the second-order term in Eq. (5) (stars), the contribution to the errors given by the Gaussian fourth-order term (second row in Eq. (5), filled circles for OU) and Gaussian plus non-Gaussian fourth-order terms (second and third row in Eq. (5), filled triangles for RTN), and the total error given by second and fourth-order terms (open circles for OU, open triangles for RTN) are also shown. Inset: the difference between gate errors due to OU and RTN processes, normalized by the gate error due to OU noise; this highlights non-Gaussian effects evidenced by Uhrig DD.

*Non-Gaussian noise* Uhrig’s dynamical control capability to suppress more efficiently the effect of second-order noise correlations makes this filter valuable for providing information on non-Gaussianity. To this end, we compare the effect of an RTN and an Ornstein-Uhlenbeck process (OU)^73^ with the same second-order statistics (and zero average value). The two processes have different higher-order statistical properties^73^. The OU, obtained as the sum of many RTN processes, is Gaussian distributed, because of the central limit theorem. The RTN is instead non-Gaussian. The error resulting from the exact numerical solution of the SSE is reported in Fig. 5 and compared with the analytical approximation Eq. (5). Open diamonds correspond to OU and filled diamonds to RTN. The error due to second-order statistics almost vanishes for $n\ge 4$, and the curves obtained by SSE are captured by the fourth-order contributions in Eq. (5).

The error due to the OU process is entirely captured by the Gaussian fourth-order term in Eq. (5), while in the error due to the RTN both Gaussian and non-Gaussian terms contribute. To highlight fourth-order statistic non-Gaussian effects in $\varepsilon$, in the inset of Fig. 5 we plot the difference between the errors due to OU (open diamonds) and RTN (filled diamonds). We observe that non-Gaussian fourth-order effects are evident already for $2n\ge 4$.

*Spatially-correlated processes* Dynamical control can also be used as a sensitive probe of noise correlations between processes affecting the two qubits, an issue whose importance emerged in recent experiments^61^. Here we consider spatially-correlated processes^74^, and assume that $z_\alpha (t)$ have the same statistical properties, $S_{z}$ and $S_{z 3}$. Under these conditions (see Supplemental Section B), spatial correlations are quantified by a single correlation coefficient^73^

9 $$\begin{aligned} \mu =\frac{S_{z_1 z_2}(\omega )}{\sqrt{S_{z_1}(\omega )S_{z_2}(\omega )}}, \end{aligned}$$

that can be detected by spectral analysis. It can be demonstrated along the same lines leading to Eq. (5) that the gate error reads

10 $$\begin{aligned} \varepsilon (\mu ) = 2(1-\mu )\Big [\varepsilon ^{[2]} + (1-\mu )\big (2 \varepsilon ^{[4]}_g + \varepsilon _{ng}^{[4]}\big )\Big ] \equiv \varepsilon ^{[2]} (\mu ) + \varepsilon _g^{[4]} (\mu )+\varepsilon _{ng}^{[4]}(\mu ). \end{aligned}$$

where the $\varepsilon ^{[2]}$, $\varepsilon _{g}^{[4]}$ and $\varepsilon ^{[4]}_{ng}$ are given in Eq. (5) where $S_\zeta$ and $S_{\zeta 3}$ are replaced by $S_{z}$ and $S_{z 3}$ respectively.

**Figure 6:**
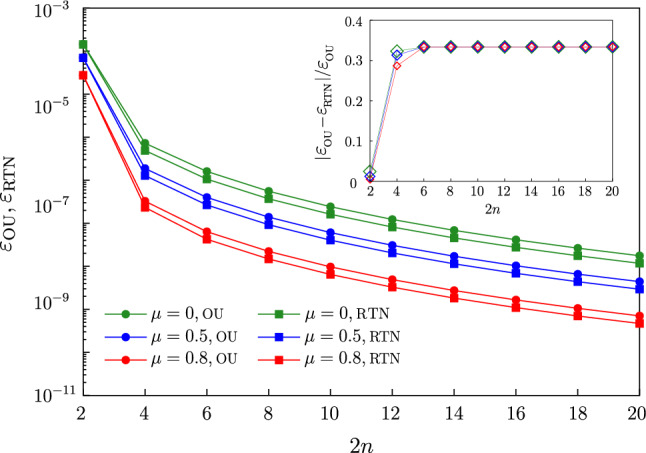
Gate error under U in the presence of Gaussian (OU) and non-Gaussian (RTN) correlated noise versus the number of pulses. The symbols are the analytical result (10) (circles for OU, squares for RTN), and lines are guides for the eye. Different colours represent different correlation coefficients $\mu =0, 0.5,0.8$ (green, blue, red). Inset: Difference between Gaussian and non-Gaussian gate errors scaled with the Gaussian error for different correlation coefficients.

In Fig. 6 we show $\varepsilon (\mu )$. The symbols (squares for OU, dots for RTN) are the analytic form Eq. (10) (reproducing the numerical solution of the SSE, not shown). For two pulses the error is due to second-order statistics $\varepsilon (\mu )\,\approx \,\varepsilon ^{[2]}(\mu )$, therefore it does not distinguish the OU process from RTN. The difference between the errors is entirely due to noise correlations entering the pre-factor $2(1-\mu )$. For larger number of pulses, the error is dominated by fourth-order statistics and non-Gaussian effects appear (difference between the squares and dots pairs for each color). It is seen that the impact of correlation depends on Gaussianity as emphasized in the inset of Fig. 6 where we plot $[\varepsilon _\text {OU}(\mu ) - \varepsilon _\text {RTN}(\mu )]/ \varepsilon _\text {OU}(\mu )$. For $2n=4$ the error is due to both second and fourth-order correlators which have a different dependence on $\mu$, for a larger number of pulses the errors are given by fourth-order correlators, resulting in a scaled difference between errors independent on $\mu$.

## Discussion

In this work, we have studied the protection of coherence by DD during processing in a non-trivial quantum gate. This is an important issue for two-qubit gates whose duration is typically much longer than single-qubit ones. To this end, we tackled the problem of selective canceling of non-commuting entries of the Hamiltonian, extending to transverse noise methods introduced for analyzing pure-dephasing longitudinal noise.

In particular, for the Ising-*xx* coupling Hamiltonian, we studied pulsed control in the presence of *local* longitudinal noise focusing on the *W*-subspace where noise is transverse to the projected Hamiltonian. From a complementary perspective, the two-qubit “principal system” may probe characteristics of environmental noise. We have shown that a QS protocol based on DD during entanglement generation may provide non-trivial information on the noise statistics, as on spatial noise correlations and/or on the fourth-order cumulant of the resulting stochastic process. Our result leverages generalized FFs describing DD while processing which differ from the standard FFs for longitudinal noise. Generalized FFs filter almost uniformly up to frequencies $~\sim \omega _c$ already with a small number of pulses yielding a very efficient protocol for QS of environmental noise.

The DD sequences we considered are routinely used in different platforms, see^10–14,18,22,30,32,33,41^. Here, we suggest a simple procedure to extract relevant information on low-frequency longitudinal noise on each qubit of an entangling gate in a fixed coupling scheme. The noise variances $\Sigma _z^2$ can be extracted from each qubit coherence, which in the presence of quasi-static pure dephasing noise decays with a peculiar Gaussian law $\rho _{+-}(t) \propto \exp {(- \Sigma _z^2 t^2)}$^75,76^, as it is observed in Ramsey experiments. Then from an entangling gate operation, the presence of spatial correlations of the noise can be checked from the gate error under the P sequence. In fact, for quasi-static noise, the analysis of Section “Gate error under dynamical control” can be extended to correlated noise leading to $\epsilon _{qs}^{(P)} \simeq \frac{\pi ^2}{2^6} 2(1-\mu ) \left( \frac{\Sigma _\zeta }{\omega _c} \right) ^2 n^{-2}$. Due to higher order filtering properties, Uhrig dynamical control on the two-qubit gate is a potential tool to distinguish quasi-static Gaussian noise, leading to an error scaling as $\epsilon _{qs}^{(U)} \propto 4(1-\mu )^2 \left( \frac{\Sigma _\zeta }{\omega _c} \right) ^4 n^{{-3.75}}$, from quasi-static non-Gaussian noise. In fact, the results in Fig. 5 for a RTN and Eq. (10) indicate that the error scales as $\epsilon \propto 2(1-\mu )^2 \left( \frac{\Sigma _\zeta }{\omega _c} \right) ^4 n^{{-3.75}}$. The quantitative distinction between the two processes requires the evaluation of the prefactors which may depend on the specific non-Gaussian process in the considered experimental setup. As a benchmark, Uhrig DD may be applied after having injected engineered low-frequency Gaussian noise into the system.

Since our results apply directly to single-qubit devices sensitive to low-frequency transverse noise, as the first generation of solid-state qubits^77^, properly designed single-qubit devices could work as quantum sensors of trispectrum if biased to make the leading noise transverse. The mapping into a single-qubit problem also suggests that there may be cases where anti-Zeno behaviour^70^ could manifest. Therefore a more complex scenario would emerge in the two-qubit dynamics where DD may be detrimental to the accuracy of entangling gates, possibly requiring quantum control and machine learning methods^78,79^.

We finally comment on the gate model we have chosen and on the relevance of different noise contributions. The *xx*-Ising interaction between qubits is a physical description of several implementations of quantum gates with fixed coupling, as capacitively or inductive coupled superconducting qubits^2^ or semiconducting qubits^65^, as well as an effective description of cavity-mediated interactions. In these cases, local longitudinal noise is potentially the major semiclassical source of dephasing^80^ which justifies our choice Eq. (1), local transverse semiclassical noise, described by the Hamiltonian $\delta {\mathscr {H}}(t) = -\frac{1}{2} x_{1}(t) \, \sigma _{1x} \otimes \mathbbm {1}_{2} - \frac{1}{2} \mathbbm {1}_{1} \otimes x_{2}(t) \, \sigma _{2x}\,$ being less relevant for our work. Indeed, low-frequency components would produce weak “transverse” dephasing between the invariant subspaces. The main effect would be leakage from the *W*-subspace, which should properly be described by a quantum noise model^81–83^ outside this work’s scope. In any case, the sequences of $\sigma _{\alpha y}$ pulses we consider tend to cancel also the $\sigma _{ \alpha x}$ coupling with the environment making the associated semiclassical noise less relevant, as we checked with SSE. Finally, noise directly affecting the *xx* qubit coupling term is not expected to be important for fixed-coupling design or for qubits coupled via a transmission line since it would be longitudinal in the *W* subspace. On the contrary, it may be non-negligible when the qubit coupling is implemented by a switchable circuit^52^.

## Methods

### Open loop control

We consider a dynamical control operated by instantaneous pulses acting locally and simultaneously on each qubit. The control sequence aims to reduce the effect of fluctuations while performing an entangling gate operation. These two requirements may be fulfilled by a sequence of an even number of simultaneous $\pi$-pulses around the *y*-axis of the Bloch sphere of each qubit which tends to average out individually all the terms of ${{{\mathscr {H}}}}(t)$ Eq. (1) but the qubit-coupling. It is described by the control Hamiltonian

11 $$\begin{aligned} {{{\mathscr {H}}}}_C(t)\,=\, {{{\mathscr {V}}}}_1(t)\otimes \mathbbm {1}_{2}\, + \,\mathbbm {1}_{1}\otimes {{{\mathscr {V}}}}_2(t), \quad \text {with} \quad {{{\mathscr {V}}}}_\alpha (t)= -i \sum _{i=1}^{2n} \delta (t-t_i) \sigma _{\alpha y} \end{aligned}$$

where the $\delta$-function approximates the process when the duration of the individual pulse is much smaller than the evolution time of the system under ${{{\mathscr {H}}}}$. Notice that the control Eq. (11) tends to suppress dynamically also local transverse noise coupled to each qubit via $\sigma _{\alpha x}$, thus we consider

12 $$\begin{aligned} \delta {\mathscr {H}}(t) = -\frac{1}{2} \Big (x_{1}(t) \, \sigma _{1x} \,+\, z_{1}(t) \, \sigma _{1z}\Big ) \otimes \mathbbm {1}_{2} - \frac{1}{2} \mathbbm {1}_{1} \otimes \Big (x_{2}(t) \, \sigma _{2x}\,+\, z_{2}(t) \, \sigma _{2z}\Big ) . \end{aligned}$$

Under these conditions, we express the density matrix $\rho (t)$ of the system as a path-integral over the realizations of the stochastic process. Denoting by $\rho \big (t | \vec \xi (t)\big )$ the density matrix for an individual realization of the stochastic process $\vec \xi (t)=\{x_1(t),z_1(t), x_2(t), z_2(t)\}$ we can express the density matrix $\rho (t)$ as a path integral over the noise realizations

13 $$\begin{aligned} \rho (t) = \int {{\mathscr {D}}}[\vec \xi (t)] \, P[\vec \xi (t)] \; \rho \big (t | \vec \xi (t)\big ) \,, \end{aligned}$$

where $P[\vec \xi (t)]$ is the probability density for the realization $\vec \xi (t)$ and ${\mathscr {D}}[\vec \xi (t)]$ contains the measure of integration^84^.

For a given realization $\vec \xi (t)$, the dynamics generated by a sequence of two pulses alternated by two Hamiltonian evolutions for a time $\Delta t_i = t_{i+1}-t_i$ is described by the propagator

14 $$\begin{aligned} {{{\mathscr {U}}}}(t_{i+1},t_{i-1}|\vec {\xi }(t))={{{\mathscr {S}}}}\,{\hat{T}}e^{-i\int _{t_i}^{t_{i+1}}{{{\mathscr {H}}}}(t^\prime )dt^\prime }\,{{{\mathscr {S}}}}\, {\hat{T}} e^{-i\int _{t_{i-1}}^{t_{i}}{{{\mathscr {H}}}}(t^\prime )dt^\prime } \,. \end{aligned}$$

where ${{\hat{T}}}$ indicates the time-ordering operator. We also have posed ${{{\mathscr {S}}}}\equiv -\sigma _{1y}\otimes \sigma _{2y}$ and ${\mathscr {H}}(t) = {\mathscr {H}}_0 + \delta {\mathscr {H}}(t)$-

For equally spaced pulses ($\Delta t_i \equiv \Delta t \ \ \forall i$) the gate operation is not altered to leading order in $\Delta t$, provided that $\Delta t \ll \min \{{\tau }_{\xi \alpha }\}$, $\tau _{\xi _\alpha }$ being the dominant (shortest) correlation time associated with the noise $\xi _\alpha (t)$. Under these conditions, the noise can be approximated, for any $t \in [t_{i-1}, t_{i+1}]$, as a static stray component^75^ $\xi (t) \approx \xi$. Consequently, the integral simply factorizes $\int _i^{i+1} {{{\mathscr {H}}}}(t^\prime ) dt^\prime \sim {{{\mathscr {H}}}}(t_i) \Delta t$. By expanding the exponential in Eq. (14) the evolution operator at the first order in $\Delta t$ reads

15 $$\begin{aligned} \begin{aligned} {{{\mathscr {U}}}}(t_{i+1},t_{i-1}|\vec {\xi }(t))&\simeq \mathbbm {1} + i \, {\omega _c} \, \Delta t \, \sigma _{1x}\otimes \sigma _{2x} \simeq e^{i\, \omega _c \Delta t\, \sigma _{1x}\otimes \sigma _{2x} } \end{aligned} \end{aligned}$$

Therefore the first order in $\Delta t$ ${{{\mathscr {U}}}}(t)$ implements a $\sqrt{i -\text {SWAP}}$ at time $t_e = 2 \Delta t$, noise effects being averaged out by the sequences of two pulses ${{{\mathscr {S}}}}$. This result extends to any sequence of an even number 2*n* of pulses such that $\sum _{k=1}^{2n} \Delta t_k= t_e \ll \min \{{\tau }_{\xi _\alpha }\}$.

We used the error $\varepsilon$, Eq. (5), in the entanglement generation protocol $|+-\rangle \rightarrow |\psi _e\rangle$ as a tool for noise sensing. Moreover, for $|z_i|<\omega _c$ the error $\varepsilon$ is close to the *W*-space infidelity thus it also quantifies the performance of DD in noisy gate processing.

Notice that while the Hamiltonian ${{{\mathscr {H}}}}_0$ operates in the proper limit a $\sqrt{i -\text {SWAP}}$ gate (see Supplemental Section A), the gate under the ${{\mathscr {S}}}$ pulse sequence tends to preserve the ideal dynamics in the *W*-subspace and not in the *Z*-subspace (both defined in Section A). This is not a problem since the unitary Eq. (15) when acting on the *Z* subspace can generate maximally entangled states. Therefore the DD sequences we consider preserve the ability of processing a perfectly entangling gate while decoupling. From the point of view of QS, the effective dynamics in the *Z*-subspace under the pulse sequences provide asymptotically information on the stochastic process $z_1 + z_2$ coupled transversally to the effective Hamiltonian.

### Dynamical control of pure dephasing correlated noise

Here we focus on local longitudinal noise, and suppose that $z_1(t)$ and $z_2(t)$ are distinct stochastic processes with a correlation degree quantified by $\mu$, Eq. (9). Control pulses ${\mathscr {S}}$ transform to $\pi$-rotation along the *z*-axis with propagator ${{{\mathscr {S}}}} = \tau _{z}$, such that $\tau _z\, {\hat{T}}e^{-\frac{i}{2}\int _{t_{k}}^{t_{k+1}} {\mathscr {H}}_g(t^\prime ) dt^\prime }\,\tau _z= {\hat{T}}e^{-\frac{i}{2}\int _{t_{k}}^{t_{k+1}} [-{\zeta }(t^\prime )\tau _x-\omega _c\tau _z]dt^\prime }$. Therefore, the effect of a control sequence can be included in the controlled-gate Hamiltonian

16 $$\begin{aligned}{} & {} {{{\mathscr {H}}}}_{cg}(t)\,=\,- \frac{{\bar{\zeta }}(t)}{2}\tau _x\,-\, \frac{\omega _c}{2} \tau _z = {{{\mathscr {H}}}}_n(t) \, +\, {{{\mathscr {H}}}}_c, \qquad {\bar{\zeta }}(t)=(-1)^{k+1} {\zeta }(t), \,\,\,t \in [t_{k-1}, t_{k}[ \end{aligned}$$

where ${{{\mathscr {H}}}}_c=-\, \frac{\omega _c}{2}\, \tau _z$ and ${{{\mathscr {H}}}}_n(t)= - \frac{1}{2} {{\bar{\zeta }}}(t) \,\tau _x$. Thus, for preparation in the single-excitation subspace, the coupled qubit evolution under local longitudinal noise and local DD is mapped to a driven pseudo-two-state system subject to transverse noise.

Introduced the propagator ${{{\mathscr {U}}}}_c(t) = e^{\frac{i}{2}\omega _c t \tau _z}$, we can write the Hamiltonian

17 $$\begin{aligned} \tilde{{{\mathscr {H}}}}_n(t)\, =\, {{{\mathscr {U}}}}_c^\dag (t) {{{\mathscr {H}}}}_n(t) {{{\mathscr {U}}}}_c(t) = -\zeta (t)\big (\tau _x \cos \omega _ct\,+\,\tau _y\sin \omega _ct\big ), \end{aligned}$$

that generate the dynamics in the “toggling” frame. This dynamics is described by $\tilde{{\mathscr {U}}} (t_e| \zeta (t))\,={\hat{T}}e^{i\int _0^{t_e}\tilde{{{\mathscr {H}}}}_n(t^\prime )dt^\prime } \,$.

The overall propagator can be written as ${{{\mathscr {U}}}}(t_e)={{{\mathscr {U}}}}_c(t_e)\tilde{{\mathscr {U}}} (t_e| \zeta (t))$ and we have $\rho (t_e) = {\mathscr {U}}(t_e) \rho (0) {\mathscr {U}}^\dagger (t_e)$. Exploiting the fact that $\rho (0) = |+-\rangle \langle +-|$ we can write the gate error as

18 $$\begin{aligned} \begin{aligned} \varepsilon =&1 - \langle \psi _e| {{{\mathscr {U}}}}(t_e)\rho (0) {{{\mathscr {U}}}}^\dagger (t_e) |\psi _e\rangle \\ =&1 - \langle \psi _e| {{{\mathscr {U}}}}_c(t_e){\tilde{\mathscr {U}}}(t_e|\zeta (t))\rho (0) {\tilde{\mathscr {U}}}^\dagger (t_e|\zeta (t)){{{\mathscr {U}}}}_c^\dagger (t_e) |\psi _e\rangle \\ =&1 - |\langle +-|\tilde{{\mathscr {U}}}(t_e|\zeta (t)) |+-\rangle |^{2} \end{aligned} \end{aligned}$$

To find analytic expressions for the gate error, we proceed analogously to^17^ and express the time propagator in the toggling frame by its Magnus expansion:

19 $$\begin{aligned} \tilde{{\mathscr {U}}} (t_e| \zeta (t))\,=\,e^{\Omega _1(t_e)+\Omega _2(t_e)+ \dots }, \end{aligned}$$

where, for simplicity, we omit the dependence of $\Omega _\alpha (t)$ on $\zeta (t)$. The first two terms of the expansion Eq. (19) read

20 $$\begin{aligned} \Omega _1(t_e)\,= & {} \,i\big [a_{1x}(t_e)\tau _x\,+\,a_{1y}(t_e)\tau _y\big ], \end{aligned}$$

21 $$\begin{aligned} \Omega _2(t_e)\,= & {} \,i\,a_{2z}(t_e)\tau _z, \end{aligned}$$

where

22 $$\begin{aligned} \begin{aligned}{}&a_{1x}(t_e) = \frac{1}{2}\int _0^{t_e} dt_1\,\zeta (t_1)\cos (\omega _ct_1) , \quad a_{1y}(t_e) = \int _0^{t_e} dt_1\,\zeta (t_1) \sin (\omega _ct_1) \,,\\&a_{2z}(t_e) = \frac{1}{4}\int _0^{t_e} dt_1 \int _0^{t_1} dt_2 \zeta (t_1)\zeta (t_2)\sin (\omega _c(t_1-t_2)),\\ \end{aligned} \end{aligned}$$

As discussed in Supplemental Section D, by truncating the Magnus expansion to the third-order the gate error can be approximated as follows

23 $$\begin{aligned} \varepsilon \simeq \varepsilon ^{[2]} + \varepsilon ^{[4]}, \end{aligned}$$

where $\varepsilon ^{[2]} = \langle a_{1y}^2\rangle$ and $\varepsilon ^{[4]} = \langle a_{2z}^2\rangle$ are of second and fourth order in the noise, respectively. The validity of these approximations is confirmed by the results presented in Section “Results”.

### Filter function formalism

The gate error in Eq. (23) can be expressed in terms of FFs of subsequent noise cumulants (for details c.f. Supplemental Section E), defined from the Uhrig filter^69^

24 $$\begin{aligned} y_n(\alpha ,t_e) \equiv 1+(-1)^{n+1}e^{i\alpha t_e}\,+\,2\sum _{k=1}^{n}(-1)^k e^{i\alpha t_k} \, \end{aligned}$$

As an example, the second-order contribution reads

25 $$\begin{aligned} \begin{aligned} \varepsilon ^{[2]}&=\, \langle \Big (\frac{i}{2}\int _0^{t_e} dt_1\,{{\bar{\zeta }}}(t_1) \sin (\omega _ct_1) \Big )^2\rangle \\&=\frac{1}{16}\int _{-\infty }^{+\infty }\frac{d\omega }{2\pi }S_{\zeta }(\omega ) \Big [\frac{\big |y_n(\omega +\omega _c,t_e)\big |^2}{(\omega +\omega _c)^2} \,+\,\frac{\big |y_n(\omega -\omega _c,t_e)\big |^2}{(\omega -\omega _c)^2}\Big ]\\&=\,\int _{-\infty }^{+\infty }\frac{d\omega }{2\pi }S_{\zeta }(\omega ) F_{1}^{}(\omega ,\omega _c,t_e,2n). \end{aligned} \end{aligned}$$

This expression defines the FF of second order

26 $$\begin{aligned} F_{1}^{}(\omega ,\omega _c,t_e,2n)=\frac{\big |y_n(\omega +\omega _c,t_e)\big |^2}{(\omega +\omega _c)^2} +\,\frac{\big |y_n(\omega -\omega _c,t_e)\big |^2}{(\omega -\omega _c)^2}. \end{aligned}$$

The same calculation for the forth order contribution reveals that $\varepsilon ^{[4]}$ can be decomposed in a Gaussian $\varepsilon _g^{[4]}$ and a non-Gaussian $\varepsilon ^{[4]}_{ng}$ contributions. Analogously to what is done for $\varepsilon ^{[2]}$, we can define two additional FFs (whose explicit expression is left to Supplemental Section E) and write

27 $$\begin{aligned} \varepsilon ^{[4]}_g\,=\,\,\int _{-\infty }^{+\infty }\frac{d\omega _1}{2\pi }\,S(\omega _1) \int _{-\infty }^{+\infty }\frac{d\omega _2}{2\pi }\,S(\omega _2)\, F_{2,g}(\vec {\omega _2},\omega _c,t_e,2n), \end{aligned}$$

and

28 $$\begin{aligned} \varepsilon ^{[4]}_{ng}\,=\, \,\int _{-\infty }^{+\infty }\frac{d\omega _1}{2\pi }\, \int _{-\infty }^{+\infty }\frac{d\omega _2}{2\pi }\, \int _{-\infty }^{+\infty }\frac{d\omega _3}{2\pi }\, S_{\zeta 3}(\vec {\omega _3}) F_{2,ng}(\vec {\omega _3},\omega _c,t_e,2n). \end{aligned}$$

and write finally Eq. (5) for the gate error.

We remark that the above expression of the gate error holds for any decoupling sequence consisting of an even number of simultaneous $\pi _y$ pulses applied at times $t_k = \delta _k t_e$ with $0< \delta _k < 1$ and $k \in \{1, 2n \}$, such that $t_{2n}=t_e$. Each sequence corresponds to different filters thus allowing either to (partly) cancel environmental effects to various orders^43^ or to filter out relevant spectral components (filtering order).

The three specific decoupling sequences, the Periodic (P), the Carr-Purcell (CP), and the Uhrig (U), discussed in this work are detailed in Section C.

### Supplementary Information

Supplementary Information.

## Data Availability

The datasets used and/or analyzed during the current study are available from the corresponding author upon reasonable request.
