# Supplementary material for: Open-loop quantum control of small-size networks for high-order cumulants and cross-correlations sensing
Source: Sci Rep. 2024 Jul 19;14:16681. doi: 10.1038/s41598-024-67503-x (PMC11271536; doi:10.1038/s41598-024-67503-x)
Supplement: Supplementary file 1 — Supplementary Information. [file 41598_2024_67503_MOESM1_ESM.pdf]

Open-loop quantum control of small-size networks for high-order cumulants and cross-correlations sensing
Supplementary information

Antonio D'Arrigo[1], Giulia Piccitto[2], Giuseppe Falci[1,3,4], and Elisabetta Paladino[1,3,4,*]

[1]Dipartimento di Fisica e Astronomia "Ettore Majorana", Università di Catania, Via Santa Sofia 64, 95123 Catania, Italy
[2]Dipartimento di Matematica e Informatica, Università di Catania, Viale Andrea Doria, 95125 Catania, Italy
[3]CNR-IMM, Catania (University unit), Consiglio Nazionale delle Ricerche, Via Santa Sofia 64, 95123 Catania, Italy
[4]Istituto Nazionale di Fisica Nucleare, Sezione di Catania, Via Santa Sofia 64, 95123 Catania, Italy
[*]elisabetta.paladino@dfa.unict.it

ABSTRACT

Here we provide supplementary information on the two-qubit entangling gate, the characterization of the stochastic processes, the application of the Magnus expansion to the considered system and the derivation of the filter functions as indicated in the main article.

A Two-qubit entangling gate

We consider two qubits labeled by $\alpha = 1,2$ living in the tensor product of the two-dimensional Hilbert space $H_1 \otimes H_2$. We define $\sigma_{\alpha k}$, with $k = x,y,z$, the Pauli matrices acting on the qubit α. The factorized computational basis $\{|\mu\nu\rangle := |\mu\rangle_1 \otimes |\nu\rangle_2\,,\ \mu,\nu = \pm\}$ is such that $\sigma_{\alpha z}|\pm\rangle_\alpha = \mp|\pm\rangle_\alpha$. The qubits are coupled by an Ising $x-x$ interaction. We considered the Hamiltonian

$$\mathscr{H} = -\frac{\Omega_1 + z_1(t)}{2}\,\sigma_{1z}\otimes \mathbb{1}_2 - \frac{\Omega_2 + z_2(t)}{2}\,\mathbb{1}_1 \otimes \sigma_{2z} + \frac{\omega_c}{2}\,\sigma_{1x}\otimes\sigma_{2x} - \frac{x_1(t)}{2}\,\sigma_{1x} - \frac{x_2(t)}{2}\,\sigma_{2x}.$$

where terms describing both longitudinal and transverse noise affecting each qubit appear. In the absence of transverse noise ($x_\alpha = 0$) the Hamiltonian $\mathscr{H}$ is block diagonal the Hilbert space being the direct sum of two invariant subspaces denoted by $W = \mathrm{span}\{|+-\rangle, |-+\rangle\}$ and $Z = \mathrm{span}\{|++\rangle, |--\rangle\}$. If the qubits are identical or if $|\Omega_1 - \Omega_2| \ll \omega_c$ we obtain an effective $\mathscr{H}_0$ with the structure of Eq. (1) of the main article where $\Omega = \Omega_1 + \Omega_2$ and $\omega_c \to \omega_c + |\Omega_1 - \Omega_2|^2/(2\omega_c)$ whose eigenvalues and eigenvectors are reported in Supplementary Table S1. This Hamiltonian implements the entanglement-generation operation studied in this work, Eq. (4) of the main article.

In the usual limit $\omega_c \ll \Omega$ the Hamiltonian in the Z subspaces presents only renormalized diagonal entries thus in this limit $\mathscr{H}_0$ can implement a gate locally equivalent to $\sqrt{\mathrm{i}-\mathrm{SWAP}}$ by evolving the system for a time $t_e = \pi/2\omega_c$.

B Characterization of the stochastic processes

We considered noise described by two stochastic processes $\xi_\alpha \equiv \{x_\alpha(t), z_\alpha(t)\}$ assumed to be 4-th order stationary and with vanishing average, $\langle \xi_\alpha(t)\rangle = 0$, where $\langle\cdot\rangle$ indicates the ensemble average. The lowest order correlation functions are $C_{\xi_\alpha}(t+\tau,t) = \langle \xi_\alpha(t+\tau)\xi_\alpha(t)\rangle \equiv C_{\xi_\alpha}(\tau)$ and the cross-covariance $C_{\xi_1\xi_2}(\tau) = \langle \xi_1(t+\tau)\xi_2(t)\rangle - \overline{\xi}_1\overline{\xi}_2$ [1]. The power spectrum $S_{\xi_\alpha}(\omega)$ and the cross-spectrum $S_{\xi_1\xi_2}(\omega)$ are given by

$$S_{\xi_\alpha}(\omega) = \int_{-\infty}^{\infty} d\tau\, C_{\xi_\alpha}(\tau)\, e^{i\omega\tau}, \qquad S_{\xi_1\xi_2}(\omega) = \int_{-\infty}^{\infty} d\tau\, C_{\xi_1\xi_2}(\tau)\, e^{i\omega\tau}. \tag{1}$$

In general, the amount of correlation between two stochastic processes $\eta_i(t)$ is quantified by the correlation factor

$$\mu = \frac{\langle[\eta_1(t) - \overline{\eta}_1][\eta_2(t) - \overline{\eta}_2]\rangle}{\sqrt{\langle[\eta_1(t) - \overline{\eta}_1]^2\rangle\langle[\eta_2(t) - \overline{\eta}_2]^2\rangle}}\,, \tag{2}$$

β	ω_β	$\lvert\beta\rangle$
0	$-\sqrt{\Omega^2+(\omega_c/2)^2}$	$-(\sin\vartheta/2)\lvert++\rangle+(\cos\vartheta/2)\lvert--\rangle$
1	$-\omega_c/2$	$\big[\lvert+-\rangle-\lvert-+\rangle\big]/\sqrt{2}$
2	$\omega_c/2$	$\big[\lvert+-\rangle+\lvert-+\rangle\big]/\sqrt{2}$
3	$\sqrt{\Omega^2+(\omega_c/2)^2}$	$\cos(\vartheta/2)\lvert++\rangle+\sin(\vartheta/2)\lvert--\rangle$

Supplementary Table 1. Eigenvalues and eigenvectors of $\mathscr{H}_0$. Here $\tan\vartheta=-\omega_c/(2\Omega)$. The two Hilbert subspaces are spanned by $\{\lvert 1\rangle,\lvert 2\rangle\}$ and $\{\lvert 0\rangle,\lvert 3\rangle\}$

where $\overline{\eta}_i \equiv \langle \eta_i(t)\rangle$. Here we assume that each component of ξ_α is the sum of independent fluctuating factors having the same variance, for instance, $z_\alpha(t)=\sum_k c_k \delta z_{\alpha,k}(t)$ where the variance of $\delta z_{\alpha,k}(t)$ does not depend on k, $\Sigma_{z,\alpha}$. This assumption is not restrictive, for instance, it models spatially-correlated processes and cross-talk effects in coupled transmons [2] or flux noise correlations between two loops of a tunable flux qubit[3] or tunable capacitively-shunted flux qubits[4], possibly due to non-local sources of flux noise or junction critical current noise. Under these conditions, the degree of correlations is expressed by the correlation coefficient relating the cross-spectrum to the individual power spectra and detectable by spectral analysis

$$S_{\xi_1\xi_2}(\omega)=\mu\sqrt{S_{\xi_1}(\omega)S_{\xi_2}(\omega)}\,. \tag{3}$$

To point out non-Gaussian effect we also evaluate the first non-vanishing higher-order correlator, i.e. the fourth-order cumulant $C^{(4)}_\xi(\vec{\tau}_3)=\langle\langle\xi(t_1)\xi(t_2)\xi(t_3)\xi(t_4)\rangle\rangle$, where $\vec{\tau}_3=(\tau_1,\tau_2,\tau_3)$ with $\tau_i=t_{i+1}-t_1$, and the trispectrum

$$S_{\xi 3}(\vec{\omega}_3)=\int_{-\infty}^{\infty} d\vec{\tau}_3 e^{-i\vec{\omega}_3\cdot\vec{\tau}_3}\,C^{(4)}_\xi(\vec{\tau}_3), \tag{4}$$

where $\vec{\omega}_3=(\omega_1,\omega_2,\omega_3)$.

C Dynamical decoupling: pulse sequences

We consider a dynamical decoupling protocol consisting of instantaneous pulses around the y axis. We divide the total evolution time t_f into $2n$ time steps, such that $t_{2n}=t_f$. The ith pulse is applied at $t_i=\delta_i t_f$, with $0\le\delta_i\le 1$. We define $\Delta t_i=t_{i+1}-t_i$ as the time interval between the ith pulse and the following one, as shown in Supplementary Fig. S1(a). Different δ_is are associated with different decoupling sequences. In the main text, we consider three different sequences, Periodic (P)[5,6], Carr-Purcell (CP)[7] and Uhrig (U)[8], such that

- $\delta_i=\frac{i}{2n}$, Periodic;
- $\delta_i=\frac{i}{2n}-\frac{1}{4n}$, Carr-Purcell;
- $\delta_i=\sin^2\left(\frac{\pi i}{2n+2}\right)$, Uhrig.

We notice that the periodic sequence with $n=1$ corresponds to the echo and that the Uhrig sequence reduces to the Carr-Purcell one for $n=2$. The Uhrig filter functions $F_1(\omega,t_e,2n)$, are given by

$$F_1(\omega,t_e,2n)=\frac{|y_n(\omega,t_e)|^2}{\omega^2}\,, \tag{5}$$

where $y_n(\omega,t_e)$ are defined in Eq. (24) in the main text. In Supplementary Fig. S1 we show Eq. (5) for the periodic (b), the Carr-Purcell (c), and the Uhrig (d) sequences.

D Magnus expansion

In this appendix, we discuss some details of the derivation of the approximations leading to Eq. (23)of the main article. To this purpose, we notice that the third term of the Magnus expansion in Eq. (19) of the main article reads

$$\Omega_3=i[a_{3x}(t_e)\tau_x+a_{3y}(t_e)\tau_y], \tag{6}$$

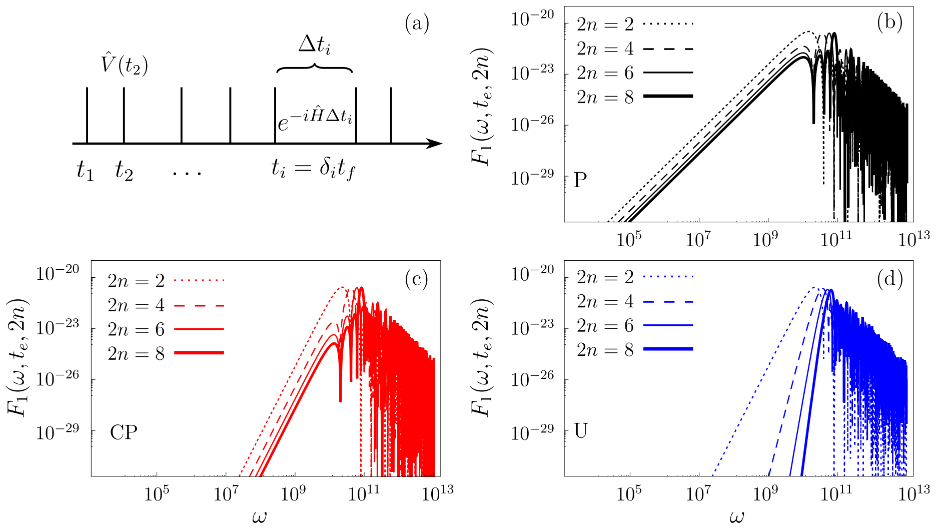

Supplementary Figure 1. Panel (a): sketch of the dynamical decoupling protocol. Panels (b), (c), (d) Plots of the second-order filters $F_1(\omega, t_e, 2n)$ for P (b), CP (c), U (d) as a function of ω. In each figure, the different line styles correspond to different numbers of applied pulses.

with

$$\begin{aligned} a_{3x}(t_e) &= \frac{1}{12}\int_0^{t_e} dt_1 \int_0^{t_1} dt_2 \int_0^{t_2} dt_3\, \zeta(t_1)\zeta(t_2)\zeta(t_3)\Big[\cos\omega_c(t_1-t_2+t_3) - \frac{1}{2}\cos\omega_c(t_1+t_2-t_3) - \cos\omega_c(t_1-t_2-t_3)\Big], \\ a_{3y}(t_e) &= \frac{1}{12}\int_0^{t_e} dt_1 \int_0^{t_1} dt_2 \int_0^{t_2} dt_3\, \zeta(t_1)\zeta(t_2)\zeta(t_3)\Big[-\sin\omega_c(t_1-t_2+t_3) + \frac{1}{2}\sin\omega_c(t_1+t_2-t_3) - \sin\omega_c(t_1-t_2-t_3)\Big]. \end{aligned} \tag{7}$$

By truncating the Magnus expansion, Eq. (19) of the main article, to the third order, the gate error at t_e reads

$$\varepsilon \simeq \langle a_{1y}^2\rangle + \langle a_{2z}^2\rangle + \langle 2a_{1y}a_{3y}\rangle - \frac{1}{3}\langle a_{1y}^4\rangle - \frac{1}{3}\langle a_{1y}^2 a_{1x}^2\rangle. \tag{8}$$

By comparing the maximum value of each contribution to the gate error, it is possible to verify that the last three terms are negligible with respect to the first two, justifying the approximation for the gate error given in Eq. (23) of the main manuscript. This is also confirmed by the exact numerical solution of the SSE for the considered pulse sequences.

E Derivation of the filter functions

In this appendix, we report the details of the derivation of the FFs contributing to the error ε. To improve the readability of this section, we derive separately the $F_1(\omega, \omega_c, t_e, 2n)$ contributing to $\varepsilon^{[2]}$ and $F_{2,g(ng)}(\omega, \omega_c, t_e, 2n)$ contributing to $\varepsilon^{[4]}_{g(ng)}$.

E.1 Derivation of $F_1(\omega, \omega_c, t_e, 2n)$

In this subsection, we derive $F_1(\omega, \omega_c, t_e, 2n)$, i.e. we explicitly write the algebra needed to go from the first to the last line of Eq. (25).

We first observe that the effect of dynamical control in the SWAP subspace is that of decomposing the time evolution as

$$\int_0^{t_e} dt_1\, \bar{\zeta}(t_1)\ldots = \sum_{k=1}^{n+1}\int_{t_{k-1}}^{t_k} dt_1\, (-1)^k \zeta(t_1)\ldots. \tag{9}$$

Let us substitute this decomposition in Eq. (25) (first line),

$$\begin{aligned} \varepsilon^{[2]} &= \langle\Big(\frac{i}{2}\int_0^{t_e} dt_1\, \bar{\zeta}(t_1)\sin(\omega_c t_1)]\Big)^2\rangle \\ &= \langle\Big(\frac{i}{2}\sum_{k=1}^{n+1}\int_{t_k-1}^{t_k} dt_1\, (-1)^k \zeta(t_1)\sin(\omega_c t_1)]\Big)^2\rangle \\ &= -\frac{1}{4}\sum_{k=1}^{n+1}\sum_{j=1}^{n+1}\int_{t_{k-1}}^{t_k} dt_1 \int_{t_{j-1}}^{t_j} dt_2\, (-1)^{k+j}\sin(\omega_c t_1)\sin(\omega_c t_2)\big\langle\zeta(t_1)\,\zeta(t_2)\big\rangle. \end{aligned} \tag{10}$$

We can use the definition

$$\langle\zeta(t_1)\zeta(t_2)\rangle = \int_{-\infty}^{+\infty}\frac{d\omega}{2\pi}\,e^{i\omega(t_1-t_2)}S_\zeta(\omega), \tag{11}$$

and write

$$\begin{aligned}\varepsilon^{[2]} &= -\frac{1}{4}\int_{-\infty}^{+\infty}\frac{d\omega}{2\pi}S_\zeta(\omega)\sum_{k,j=1}^{n+1}(-1)^{k+j}\int_{t_{k-1}}^{t_k}dt_1\int_{t_{j-1}}^{t_j}dt_2\,\sin(\omega_c t_1)\sin(\omega_c t_2)\,e^{i\omega(t_1-t_2)}\\ &= -\frac{1}{4}\int_{-\infty}^{+\infty}\frac{d\omega}{2\pi}S_\zeta(\omega)\sum_{k,j=1}^{n+1}(-1)^{k+j}\int_{t_{k-1}}^{t_k}dt_1\int_{t_{j-1}}^{t_j}dt_2\Big[\cos[\omega_c(t_1-t_2)]-\cos[\omega_c(t_1+t_2)]\Big]\,e^{i\omega(t_1-t_2)},\end{aligned} \tag{12}$$

where we have used the relation

$$\cos[\omega_c(t_1\mp t_2)] = \cos(\omega_c t_1)\cos(\omega_c t_2)\pm\sin(\omega_c t_1)\sin(\omega_c t_2). \tag{13}$$

Let us focus on the first contribution to the time integrals in the last row of Eq. (12)

$$\begin{aligned}&\sum_{k,j=1}^{n+1}(-1)^{k+j}\int_{t_{k-1}}^{t_k}dt_1\int_{t_{j-1}}^{t_j}dt_2\,\cos[\omega_c(t_1-t_2)]\,e^{i\omega(t_1-t_2)}\\ &= \frac{1}{2}\sum_{k,j=1}^{n+1}(-1)^{k+j}\int_{t_{k-1}}^{t_k}dt_1\int_{t_{j-1}}^{t_j}dt_2\,\left(e^{i\omega_c(t_1-t_2)}+e^{-i\omega_c(t_1-t_2)}\right)e^{i\omega(t_1-t_2)}\\ &= \frac{1}{2}\sum_{k,j=1}^{n+1}(-1)^{k+j}\left[\int_{t_{k-1}}^{t_k}dt_1\,e^{i(\omega+\omega_c)t_1}\int_{t_{j-1}}^{t_j}dt_2\,e^{-i(\omega+\omega_c)t_2}+\int_{t_{k-1}}^{t_k}dt_1\,e^{i(\omega-\omega_c)t_1}\int_{t_{j-1}}^{t_j}dt_2\,e^{-i(\omega-\omega_c)t_2}\right].\end{aligned} \tag{14}$$

We notice that

$$\sum_{k=1}^{n+1}(-1)^k\int_{t_{k-1}}^{t_k}dt_1\,e^{i\alpha t_1} = \frac{1}{i\alpha}\left[1+(-1)^{n+1}e^{i\alpha t_e}+2\sum_{k=1}^{n}(-1)^k e^{i\alpha t_k}\right]\equiv\frac{1}{i\alpha}y_n(\alpha,t_e), \tag{15}$$

being $y_n(\alpha,t_e)$ the Uhrig filter introduced in Eq. (24). We can thus substitute and obtain

$$\begin{aligned}&\sum_{k,j=1}^{n+1}(-1)^{k+j}\int_{t_{k-1}}^{t_k}dt_1\int_{t_{j-1}}^{t_j}dt_2\,\cos[\omega_c(t_1-t_2)]\,e^{i\omega(t_1-t_2)}\\ &= \frac{1}{2}\left[\frac{\left|y_n(\omega+\omega_c,t_e)\right|^2}{(\omega+\omega_c)^2}+\frac{\left|y_n(\omega-\omega_c,t_e)\right|^2}{(\omega-\omega_c)^2}\right].\end{aligned} \tag{16}$$

Analogous calculations for the contribution due to $\cos[\omega_c(t_1+t_2)]$ lead to

$$\sum_{k,j=1}^{n+1}(-1)^{k+j}\int_{t_{k-1}}^{t_k}dt_1\int_{t_{j-1}}^{t_j}dt_2\,\cos\omega_c(t_1+t_2)\,e^{i\omega(t_1-t_2)} = \frac{\Re[y_n(\omega+\omega_c,t_e)\,y_n^*(\omega-\omega_c,t_e)]}{\omega^2-\omega_c^2}. \tag{17}$$

However, we checked that this contribution is vanishing for all the considered sequences. Combining all these terms we obtain obtain Eq. (25).

E.2 Derivation of $F_{2,g}(\omega,\omega_c,t_e,2n)$ and $F_{2,ng}(\omega,\omega_c,t_e,2n)$

To evaluate the FF in Eq. (26) we observe that

$$\int_0^{t_e}dt_1\int_0^{t_1}dt_2 = \sum_{k=2}^{n+1}\int_{t_{k-1}}^{t_k}dt_1\sum_{m=1}^{k-1}\int_{t_{m-1}}^{t_m}dt_2+\sum_{k=1}^{n+1}\int_{t_{k-1}}^{t_k}dt_1\int_{t_{k-1}}^{t_1}dt_2. \tag{18}$$

It is convenient to introduce the two-times integral operator

$$\mathscr{A}^{[2]}(t_1,t_2) = \frac{1}{4}\left(\sum_{k=2}^{n+1}\sum_{m=1}^{k-1}(-1)^{k+m}\int_{t_{k-1}}^{t_k}dt_1\int_{t_{m-1}}^{t_m}dt_2+\sum_{k=1}^{n+1}\int_{t_{k-1}}^{t_k}dt_1\int_{t_{k-1}}^{t_1}dt_2\right), \tag{19}$$

and the four-time integral operator

$$\mathscr{A} = \mathscr{A}^{[2]}(t_1,t_2) \times \mathscr{A}^{[2]}(t_3,t_4) \tag{20}$$

Therefore we can write

$$\begin{aligned}
\varepsilon^{[4]} &= \left\langle \left(\frac{i}{4} \int_0^{t_e} dt_1 \int_0^{t_1} dt_2\, \bar{\zeta}(t_1)\bar{\zeta}(t_2) \sin[\omega_c(t_1-t_2)] \right)^2 \right\rangle \\
&= \frac{1}{16} \int_0^{t_e} dt_1 \int_0^{t_1} dt_2 \int_0^{t_e} dt_3 \int_0^{t_3} dt_4 \langle \bar{\zeta}(t_1)\bar{\zeta}(t_2)\bar{\zeta}(t_3)\bar{\zeta}(t_4)\rangle \sin[\omega_c(t_1-t_2)] \sin[\omega_c(t_3-t_4)] \\
&= \mathscr{A}\Big\{ \langle \zeta(t_1)\zeta(t_2)\zeta(t_3)\zeta(t_4)\rangle \sin[\omega_c(t_1-t_2)] \sin[\omega_c(t_3-t_4)] \Big\},
\end{aligned}$$

The average $\langle \zeta(t_1)\zeta(t_2)\zeta(t_3)\zeta(t_4)\rangle$ can be decomposed in a Gaussian and a non-Gaussian contributions

$$\langle \zeta(t_1)\zeta(t_2)\zeta(t_3)\zeta(t_4)\rangle = \langle \zeta(t_1)\zeta(t_2)\zeta(t_3)\zeta(t_4)\rangle_g + \langle \zeta(t_1)\zeta(t_2)\zeta(t_3)\zeta(t_4)\rangle_{ng}, \tag{21}$$

the former being expressed by the product of two-point correlation functions, the latter being the fourth-order cumulant. In details, the Gaussian contribution reads

$$\begin{aligned}
\langle \zeta(t_1)\zeta(t_2)\zeta(t_3)\zeta(t_4)\rangle_g &= \langle \zeta(t_1)\zeta(t_2)\rangle\langle \zeta(t_3)\zeta(t_4)\rangle + \langle \zeta(t_1)\zeta(t_3)\rangle\langle \zeta(t_2)\zeta(t_4)\rangle + \langle \zeta(t_1)\zeta(t_4)\rangle\langle \zeta(t_2)\zeta(t_3)\rangle \\
&= \int_{-\infty}^{+\infty} \frac{d\omega_1}{2\pi} S_\zeta(\omega_1) \int_{-\infty}^{+\infty} \frac{d\omega_2}{2\pi} S_\zeta(\omega_2) e^{i\omega_1(t_1-t_2)} e^{i\omega_2(t_3-t_4)} \\
&= \int_{-\infty}^{+\infty} \frac{d\omega_1}{2\pi} S_\zeta(\omega_1) \int_{-\infty}^{+\infty} \frac{d\omega_2}{2\pi} S_\zeta(\omega_2) e^{i\omega_1(t_1-t_3)} e^{i\omega_2(t_2-t_4)} \\
&= \int_{-\infty}^{+\infty} \frac{d\omega_1}{2\pi} S_\zeta(\omega_1) \int_{-\infty}^{+\infty} \frac{d\omega_2}{2\pi} S_\zeta(\omega_2) e^{i\omega_1(t_1-t_4)} e^{i\omega_2(t_2-t_3)}
\end{aligned} \tag{22}$$

while the non-Gaussian

$$\langle \zeta(t_1)\zeta(t_2)\zeta(t_3)\zeta(t_4)\rangle_{ng} = \int_{-\infty}^{+\infty} \frac{d\omega_1}{2\pi} e^{i\omega_1(t_2-t_1)} \int_{-\infty}^{+\infty} \frac{d\omega_2}{2\pi} \mathrm{e}^{i\omega_2(t_3-t_1)} \int_{-\infty}^{+\infty} \frac{d\omega_3}{2\pi} e^{i\omega_3(t_4-t_1)} S_{\zeta 3}(\omega_1,\omega_2,\omega_3), \tag{23}$$

being $S_{\zeta 3}(\omega_1,\omega_2,\omega_3)$ the trispectrum (4).

Through these expressions, we can define the Gaussian and the non-Gaussian contribution to the gate error

$$\begin{aligned}
\varepsilon_g^{[4]} &= \mathscr{A}\Big\{ \langle \zeta(t_1)\zeta(t_2)\zeta(t_3)\zeta(t_4)\rangle_g \sin[\omega_c(t_1-t_2)] \sin[\omega_c(t_3-t_4)] \Big\} \\
\varepsilon_{ng}^{[4]} &= \mathscr{A}\Big\{ \langle \zeta(t_1)\zeta(t_2)\zeta(t_3)\zeta(t_4)\rangle_{ng} \sin[\omega_c(t_1-t_2)] \sin[\omega_c(t_3-t_4)] \Big\}.
\end{aligned} \tag{24}$$

We rewrite

$$\begin{aligned}
&\sin[\omega_c(t_1-t_2)] \sin[\omega_c(t_3-t_4)] = \cos[\omega_c(t_1-t_2-t_3+t_4)] - \cos[\omega_c(t_1-t_2+t_3-t_4)] \\
&= \frac{1}{2}\left[e^{i\omega_c(t_1-t_2-t_3+t_4)} + e^{-i\omega_c(t_1-t_2-t_3+t_4)} - e^{i\omega_c(t_1-t_2+t_3-t_4)} - e^{-i\omega_c(t_1-t_2+t_3-t_4)} \right].
\end{aligned} \tag{25}$$

We can now operate analogously to what was done in the previous section and rewrite (we skip the details of the lengthy calculations). For simplicity, we introduce the function

$$\begin{aligned}
&\chi_n(\alpha,\beta,t_e) \equiv \mathscr{A}^{[2]}(t_1,t_2)\{e^{i\alpha t_1} e^{i\beta t_2}\} \\
&= (-1)^{n+2} \frac{e^{i\alpha t_e}}{\alpha\beta} y_n(\beta,t_e) - 2\sum_{k=1}^{n} (-1)^k \frac{e^{i\alpha t_k}}{\alpha\beta} \tilde{y}_{n,k}(\beta,t_e) + \frac{e^{i(\alpha+\beta)t_e}-1}{\alpha(\alpha+\beta)},
\end{aligned} \tag{26}$$

where

$$\tilde{y}_{n,k}(\beta,t_e) = 1 + (-1)^{k+1} e^{i\beta t_k} + 2\sum_{m=1}^{k} (-1)^m e^{i\beta t_m}, \tag{27}$$

being the t_ks the same entering in $y_n(\beta,t_e)$. With this notation, we can write

$$\mathscr{A}\Big\{ e^{i\alpha t_1} e^{i\beta t_2} e^{i\gamma t_3} e^{i\delta t_4} \Big\} = \chi_n(\alpha,\beta,t_e)\chi_n(\gamma,\delta,t_e). \tag{28}$$

Collecting these results we obtain

$$\begin{aligned}
\varepsilon_g^{[4]} &= \int_{-\infty}^{+\infty} \frac{d\omega_1}{2\pi} S(\omega_1) \int_{-\infty}^{+\infty} \frac{d\omega_2}{2\pi} S(\omega_2) F_{2,g}(\omega_1, \omega_2, \omega_c, t_e, 2n), \\
\varepsilon_{ng}^{[4]} &= \int_{-\infty}^{+\infty} \frac{d\omega_1}{2\pi} \int_{-\infty}^{+\infty} \frac{d\omega_2}{2\pi} \int_{-\infty}^{+\infty} \frac{d\omega_3}{2\pi} S_{\zeta 3}(\omega_1, \omega_2, \omega_3) F_{2,ng}(\omega_1, \omega_2, \omega_3, \omega_c, t_e, 2n),
\end{aligned} \tag{29}$$

which defines the fourth-order Gaussian and non-Gaussian FFs $F_{2,g}(\omega_1, \omega_2, \omega_c, t_e, 2n)$ and $F_{2,ng}(\omega_1, \omega_2, \omega_3, \omega_c, t_e, 2n)$, that can be written as

$$\begin{aligned}
F_{2,g}(\vec{\omega}_2, \omega_c) = \frac{1}{2^6} \Big\{ & |\chi_n(\omega_1 + \omega_c, \omega_2 - \omega_c, t_e)|^2 + |\chi_n(\omega_1 - \omega_c, \omega_2 + \omega_c, t_e)|^2 \\
& + 2\Re\big[\chi_n(\omega_1 + \omega_c, -\omega_1 - \omega_c, t_e)\chi_n(\omega_2 - \omega_c, -\omega_2 + \omega_c, t_e) \\
& \quad + \chi_n(\omega_1 + \omega_c, -\omega_2 - \omega_c, t_e)\chi_n(-\omega_2 - \omega_c, -\omega_1 + \omega_c, t_e) \\
& \quad - \chi_n(\omega_1 + \omega_c, -\omega_1 - \omega_c, t_e)\chi_n(\omega_2 + \omega_c, -\omega_2 - \omega_c, t_e) \\
& \quad - \chi_n(\omega_1 + \omega_c, \omega_2 - \omega_c, t_e)\chi_n(-\omega_1 + \omega_c, -\omega_2 - \omega_c, t_e) \\
& \quad - \chi_n(\omega_1 + \omega_c, \omega_2 - \omega_c, t_e)\chi_n(-\omega_2 + \omega_c, -\omega_1 - \omega_c, t_e), \big] \Big\},
\end{aligned} \tag{30}$$

and

$$\begin{aligned}
F_{2,ng}(\vec{\omega}_3, \omega_c, t_e, 2n) = \frac{1}{2^6} \Big\{ & -\chi_{2n}(\omega_c - \omega_1 - \omega_2 - \omega_3, -\omega_c + \omega_1, t_e)\, \chi_{2n}(\omega_c + \omega_2, -\omega_c + \omega_3, t_e) \\
& + \chi_{2n}(\omega_c - \omega_1 - \omega_2 - \omega_3, -\omega_c + \omega_1, t_e)\, \chi_{2n}(-\omega_c + \omega_2, \omega_c + \omega_3, t_e) \\
& + \chi_{2n}(\omega_c - \omega_1 - \omega_2 - \omega_3, \omega_c + \omega_1, t_e)\, \chi_{2n}(\omega_c + \omega_2, -\omega_c + \omega_3, t_e) \\
& - \chi_{2n}(\omega_c - \omega_1 - \omega_2 - \omega_3, \omega_c + \omega_1, t_e)\, \chi_{2n}(-\omega_c + \omega_2, \omega_c + \omega_3, t_e) \Big\}.
\end{aligned} \tag{31}$$

References

1. Papoulis, A. & Unnikrishna Pillai, S. *Probability, Random Variables and Stochastic Processes* (McGraw-Hill Inc.,US, 1984).
2. D'Arrigo, A., Mastellone, A., Paladino, E. & Falci, G. Effects of low-frequency noise cross-correlations in coupled superconducting qubits. *New Jour. Phys.* **10**, DOI: 10.1088/1367-2630/10/11/115006 (2008).
3. Gustavsson, S. *et al.* Dynamical decoupling and dephasing in interacting two-level systems. *Phys. Rev. Lett.* **109**, 010502, DOI: 10.1103/PhysRevLett.109.010502 (2012).
4. Trappen, R. *et al.* Decoherence of a tunable capacitively shunted flux qubit (2023). 2307.13961.
5. Viola, L. & Lloyd, S. Dynamical suppression of decoherence in two-state quantum systems. *Phys. Rev. A* **58**, 2733–2744, DOI: 10.1103/PhysRevA.58.2733 (1998).
6. Viola, L., Knill, E. & Lloyd, S. Dynamical decoupling of open quantum systems. *Phys. Rev. Lett.* **82**, 2417–2421, DOI: 10.1103/PhysRevLett.82.2417 (1999).
7. Carr, H. Y. & Purcell, E. M. Effects of diffusion on free precession in nuclear magnetic resonance experiments. *Phys. Rev.* **94**, 630, DOI: 10.1103/PHYSREV.94.630 (1954).
8. Uhrig, G. S. Keeping a quantum bit alive by optimized π-pulse sequences. *Phys. Rev. Lett.* **98**, 100504, DOI: 10.1103/PhysRevLett.98.100504 (2007).
